# The association between air pollution and preterm birth and low birth weight in Guangdong, China

**DOI:** 10.1186/s12889-018-6307-7

**Published:** 2019-01-03

**Authors:** Ying Liu, Jihong Xu, Dian Chen, Pei Sun, Xu Ma

**Affiliations:** 1 Institute of Psychology Continuing Education College, University of the Chinese Academy of Sciences, National Research Institute for Family Planning, No.12, Dahuisi Road, Hai Dian District, Beijing, 100081 China; 2 Research Center for Mental Health and Behavior Big Data, National Research Institute for Family Planning, Beijing, China; 3 Department of Psychology, Tsinghua University, Beijing, China

**Keywords:** Air pollution, Preterm birth, Low birth weight

## Abstract

**Background:**

A mountain of evidence has shown that people’s physical and mental health can be affected by various air pollutions. Poor pregnancy outcomes are associated with exposure to air pollution. Therefore, this study aims to investigate the association between air pollutions (PM_2.5_, PM_10_, SO_2_, NO_2_, CO, and O_3_) and preterm birth/low birth weight in Guangdong province, China.

**Method:**

All maternal data and birth data from January 1, 2014 to December 31, 2015 were selected from a National Free Pre-pregnancy Check-ups system, and the daily air quality data of Guangdong Province was collected from China National Environmental Monitoring Center. 1784 women with either preterm birth information (*n* = 687) or low birth weight information (*n* = 1097) were used as experimental group. Control group included 1766 women with healthy birth information. Logistic regression models were employed to evaluate the effects of air pollutants on the risk of preterm birth and low birth weight.

**Results:**

The pollution levels of PM_2.5_, PM_10_, SO_2_, NO_2_, CO, and O_3_ in Guangdong province were all lower than the national air pollution concentrations. The concentrations of PM_2.5_, PM_10_, SO_2_, NO_2_ and CO had obvious seasonal trends with the highest in winter and the lowest in summer. O_3_ concentrations in September (65.72 μg/m^3^) and October (84.18 μg/m^3^) were relatively higher. After controlling for the impact of confounding factors, the increases in the risk of preterm birth were associated with each 10 μg/m^3^ increase in PM_2.5_ (OR 1.043, 95% CI 1.01–1.09) and PM_10_ (OR 1.039, 95% CI 1.01~1.14) during the first trimester and in PM_2.5_ (OR 1.038, 95% CI 1.01~1.12), PM_10_ (OR 1.024, 95% CI 1.02~1.09), SO_2_ (OR 1.081, 95% CI 1.01~1.29), and O_3_ (OR 1.016, 95% CI 1.004~1.35) during the third trimester. The increase in the risk of low birth weight was associated with PM_2.5_, PM_10_, NO_2_, and O_3_ in the first month and the last month.

**Conclusion:**

This study provides further evidence for the relationships between air pollutions and preterm birth/low birth weight. Pregnant women are recommended to reduce or avoid exposure to air pollutions during pregnancy, especially in the early and late stages of pregnancy.

## Background

An enormous body of evidence has shown that people’s physical and mental health can be affected by various air pollutions [1–3]. More recently, an increasing number of researches have shown that there is a potential association between the exposure to air pollution and poor pregnancy outcomes, such as preterm birth, low birth weight, and mortality [4–6]. A review from Stieb et al. (2012) examined the association between air pollution and low birth, change in birth weight and preterm birth for pollutants including particulate matter < 10 and 2.5 μm in aerodynamic diameter (PM_10_ and PM_2.5_), nitrogen dioxide (NO_2_), sulphur dioxide (SO_2_), and carbon monoxide (CO) [7]. Xu et al. (1995) found that in the third trimester, the duration of gestation was significantly reduced with the increase in levels of sulfur dioxide (SO_2_) and total suspended particle (TSP) [8]. The risk of low birth weight increased as the mothers were exposed to higher levels of pollutants in the first trimester in Seoul [9]. Another recent meta-analysis study has also showed that maternal exposure to fine particulate air pollution increases the risk of preterm birth and term low birth weight [10]. The researchers indicated that exposure to high concentrations of PM_2.5_ in the second trimester [11] and exposure to PM_10_ in the late pregnancy [12] had a strong effect on preterm birth, while birth weight was more consistently correlated to maternal exposure to PM_2.5_ than preterm birth [13]. Although some studies found that air pollutants significantly impacted birth outcomes, others failed to find such associations, leading to inconsistent and controversial conclusions [4].

In China, the air quality in urban and rural areas has deteriorated in recent years. The Chinese government has paid great attention to the environmental protection issues, such as the average concentration limits of PM and ozone (O_3_) are included in the “National Ambient Air Quality Standards” implemented by the Chinese Ministry of Environmental Protection in 2016, and the concentration limits of PM and NO_2_ are also adjusted. Data from the National Monitoring Center shown that the annual average concentrations of PM_10_, PM_2.5_, and SO_2_ have decreased, whereas the pollution levels of NO_2_ and O_3_ have increased. [14] It is worth noting that the concentration of O_3_ is increasing year by year, and O_3_ pollution has gradually replaced PM_2.5_ as the primary air pollutant in major Chinese cities [14]. However, most previous studies have only focused on PM. Not all previous studies have found that O_3_ is a risk factor for preterm birth [11], and few studies have focused on the association between O_3_ and low birth weight.

Therefore, the objective of this study is to respectively investigate the relationships between atmospheric pollutants (i.e., PM_2.5_, PM_10_, SO_2_, NO_2_, CO, O_3_) and preterm birth/low birth weight in Guangdong province, a main province located in the southern China. The expected results are that there are significant correlations between the incidence of preterm birth/low birth weight and maternal exposure to air pollutions, respectively.

## Methods

### Data

All maternal data and birth data from January 1, 2014 to December 31, 2015 were selected from a National Free Pre-pregnancy Check-ups (NFPC) system. The NFPC has been supported by the National Health and Family Planning Commission of the People’s Republic of China since 2010, a population-based health survey for couples of reproductive-aged who wish to conceive. In this study, maternal demographic information (e.g. maternal age, education level, occupation, registered residence, pregnancy time and gestational age), pregnancy outcomes (e.g. preterm birth weight and low birth weight) and infant information (e.g. infant sex, birth weight, childbirth time, and parity) were collected. A total of 86,139 reproductive-aged women with fetal information were selected. Of these women, 1784 had either preterm birth information (*n* = 687) or low birth weight information (*n* = 1097), and 84,095 had healthy birth information excluding the data of duplicate records. The control group was selected from the 84,095 reproductive-aged women with healthy birth information using the simple random sampling method. To match the number of women in the experimental group, 2.1% of the data with healthy birth information were randomly selected as the control group (*N* = 1766). The participants were from 20 to 49 years old with the average age of 28.45 ± 4.53 years. The majority of the participants belonged to Han ethnic group (96.19%). There were no significant differences in demographic variables between the experimental group and the control group (*p* > 0.05). The descriptive statistics regarding the participants were showed in Table 1. It should be noted that preterm birth here is defined as the live birth of a baby between 28 and 37 weeks of gestational age, and low birth weight is defined as the live birth weight of baby less than 2500 g [15].

**Table 1:** Descriptive statistics of the participants

	Experimental group		Control group		*χ* ^2^	*p*
	*N*	%	*N*	%		
Age^a^					912.29	0.38
20~25	350	25.38	368	26.82		
26~30	690	50.04	674	49.13		
31~35	237	17.19	239	17.42		
36~40	73	5.29	60	4.37		
41~49	29	2.10	31	2.26		
Education^a^					13.67	0.85
Primary school and below	20	1.71	18	1.59		
Junior high school	271	23.20	319	28.21		
Senior high school	286	24.49	283	25.02		
College	526	45.03	473	41.82		
Postgraduate and above	65	5.57	38	3.36		
Occupation^a^					39.95	0.52
Farmer	249	22.02	266	24.01		
Worker	223	19.72	259	23.38		
Service industry	111	9.81	102	9.21		
Business	41	3.63	47	4.24		
Housework	33	2.92	23	2.08		
Teacher/Civil servant	395	34.92	323	29.18		
Others	79	6.98	88	7.94		
Registered residence^a^					1.19	0.28
Rural	845	60.97	912	66.23		
Urban	541	39.03	465	33.77		

Note: ^a^ Variables with missing data

On the other hand, the air pollution data was collected from China National Environmental Monitoring Center which provides daily concentrations of pollutants from 111 monitoring site stations in Guangdong province, including 102 National Ambient Air Quality Monitoring Sites in 21 prefecture-level cities and Shunde District, 8 regional stations and 1 superstation. There are typically multiple monitors located within a city, some of which provide integrated daily measurements. Therefore, city-specific exposure analysis can be used to reduce exposure misclassification. The routine detections of pollutants mainly include the detections of PM_2.5_, PM_10_, SO_2_, NO_2_, CO, and O_3_. The 24-h average concentrations of PM_2.5_, PM_10_, SO_2_, NO_2_, CO, and 8-h (from 10 AM to 6 AM) average concentration of O_3_ were collected. There must be at least 75% of the one-hour values on a particular day in order to calculate the 24-h average concentration of PM_2.5_, PM_10_, SO_2_, NO_2_, and CO. It is required to have at least six hourly values from 10 AM to 6 PM to calculate the 8-h average of O_3_ [16].

### Statistical analyses

Excel 2010, SPSS 20.0, and some packages in R 3.5.1 (i.e., ‘rms’, ‘Hmisc’, ‘lrm’, and ‘mgcv’) were used for data analysis. The mean values of the concentrations of air pollutants measured at all monitors in each city were used as the daily air pollution levels. Logistic regression models were employed to evaluate the effects of air pollutants on the risks of preterm birth and low birth weight, which effectively controlled for the impact of other variables such as maternal age, education level, occupation, registered residence, gestational age, infant sex, childbirth time, month of conception and parity. According to the division of seasons by meteorological department, this study divided the whole year into four seasons: spring (from March to May), summer (from June to August), autumn (from September to November) and winter (from December to February).

Natural cubic splines were employed for air pollutants in single-pollutant model to check whether the associations between air pollutants and preterm birth/low birth weight were linear or nonlinear. The degree of freedom (*df*) was selected by assessing the model fitting on the basis of the Akaike Information Criterion (AIC). If the relationships between air pollution and preterm birth/low birth weight were linear, then the odds ratios (ORs) and the corresponding 95% confidence intervals (CIs) of preterm birth/low birth weight for a 10 μg/m^3^ increase in PM_2.5_, PM_10_, SO_2_, NO_2_, O_3_ and for a 100 μg/m^3^ increase in CO were calculated; Each air pollutant was added into the single-pollutant model separately. Otherwise, the ORs and the 95% CIs of preterm birth/low birth weight comparing the 75th and 95th percentiles of air pollution versus the minimum preterm birth/low birth weight concentration of air pollution (threshold) were computed. To determine the threshold of air pollutant, we plotted the relationships between air pollutants and preterm birth/low birth weight, and then visually examined the possible range of the threshold. The concentrations of air pollutants corresponding to the lowest AIC values were selected as the thresholds (minimum preterm birth/low birth weight concentrations of air pollutants) [17]. In addition, the pregnancy period was divided into three stages, called trimesters: first trimester (from the first month to the third month), second trimester (from the fourth month to the seventh month), and third trimester (from the eighth month to birth). Statistical inferences were based on the significance level of 0.05 (i.e., *p* < 0.05).

## Results

Table 2 provides the descriptive statistics for the daily number of air pollution concentrations. The mean concentrations of PM_2.5_, PM_10_, SO_2_, NO_2_, CO, and O_3_ were 36.45 μg/m^3^, 55.45 μg/m^3^, 14.90 μg/m^3^, 26.37 μg/m^3^, 1.02 mg/m^3^, and 56.40 μg/m^3^, respectively. According to the reference values of National Ambient Air Quality Standard (GB 3095–2012), the pollution levels of PM_2.5_, PM_10_, SO_2_, NO_2_, CO, and O_3_ in Guangdong province were all lower than the national air pollution concentrations.

**Table 2:** Descriptive statistics for the daily number of air pollution concentrations

Pollutant (unit)	$\overline{x}$±*s*	*P* (25)	Median	*P* (75)	Range	Concentration limits
PM_2.5_ (μg/m^3^)	36.45 ± 18.54	22.00	33.00	47.00	11.00~126.00	75
PM_10_ (μg/m^3^)	55.45 ± 24.56	36.00	50.00	69.00	16.00~171.00	150
SO_2_ (μg/m^3^)	14.90 ± 5.35	11.00	14.00	18.00	6.00~42.00	150
NO_2_ (μg/m^3^)	26.37 ± 10.09	19.00	24.00	31.00	10.00~72.00	80
CO (mg/m^3^)	1.02 ± 0.21	0.87	0.97	1.16	0.66~1.85	4
O_3_ (μg/m^3^)	56.40 ± 19.11	42.00	52.00	68.00	17.00~123.00	160

*Note*: Concentration limits of PM_2.5_, PM_10_, SO_2_, NO_2_ and CO: the maximum allowable value of the average concentration within any 24 h. Concentration limit of O_3_: the maximum allowable value of the average concentration within any 8 h

**Fig. 1:**
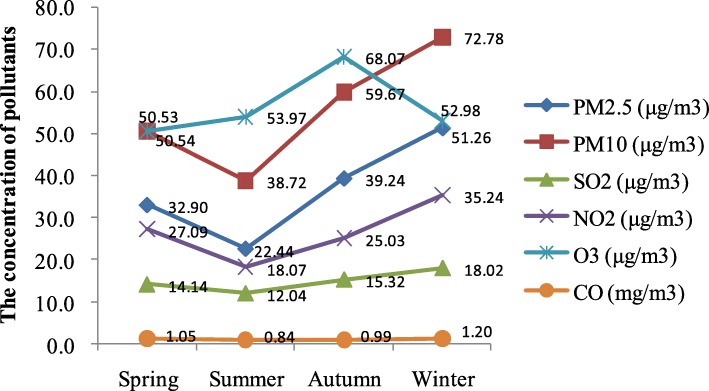
Graphical illustration of the seasonal distribution of air pollution

Table 3 and Fig. 1 present the seasonal distributions of air pollution concentrations. The results showed that the concentrations of PM_2.5_, PM_10_, SO_2_, NO_2_ and CO had obvious seasonal trends with the highest in winter and the lowest in summer. However, the concentration of O_3_ mainly concentrated in autumn, especially on October (84.18 μg/m^3^) and September (65.72 μg/m^3^).

**Table 3:** Seasonal distribution of air pollution

Seasons	Month	PM_2.5_ (μg/m^3^)	PM_10_ (μg/m^3^)	SO_2_ (μg/m^3^)	NO_2_ (μg/m^3^)	CO (mg/m^3^)	O_3_ (μg/m^3^)
Spring	3	39.07	57.47	15.51	31.28	1.14	43.17
	4	35.49	54.48	14.77	26.80	1.05	59.98
	5	24.13	39.66	12.15	23.18	0.96	48.45
Summer	6	20.63	36.97	11.32	18.50	0.84	50.32
	7	23.15	39.42	11.58	17.13	0.85	56.71
	8	23.55	39.78	13.21	18.59	0.84	54.88
Autumn	9	29.63	46.25	12.90	19.92	0.91	65.72
	10	47.82	72.16	17.18	26.44	1.01	84.18
	11	40.27	60.60	15.87	28.73	1.06	54.30
Winter	12	41.15	62.21	17.85	33.73	1.08	44.50
	1	66.24	93.57	22.40	43.53	1.34	61.65
	2	46.38	62.55	13.80	28.46	1.18	52.80

**Fig. 2:**
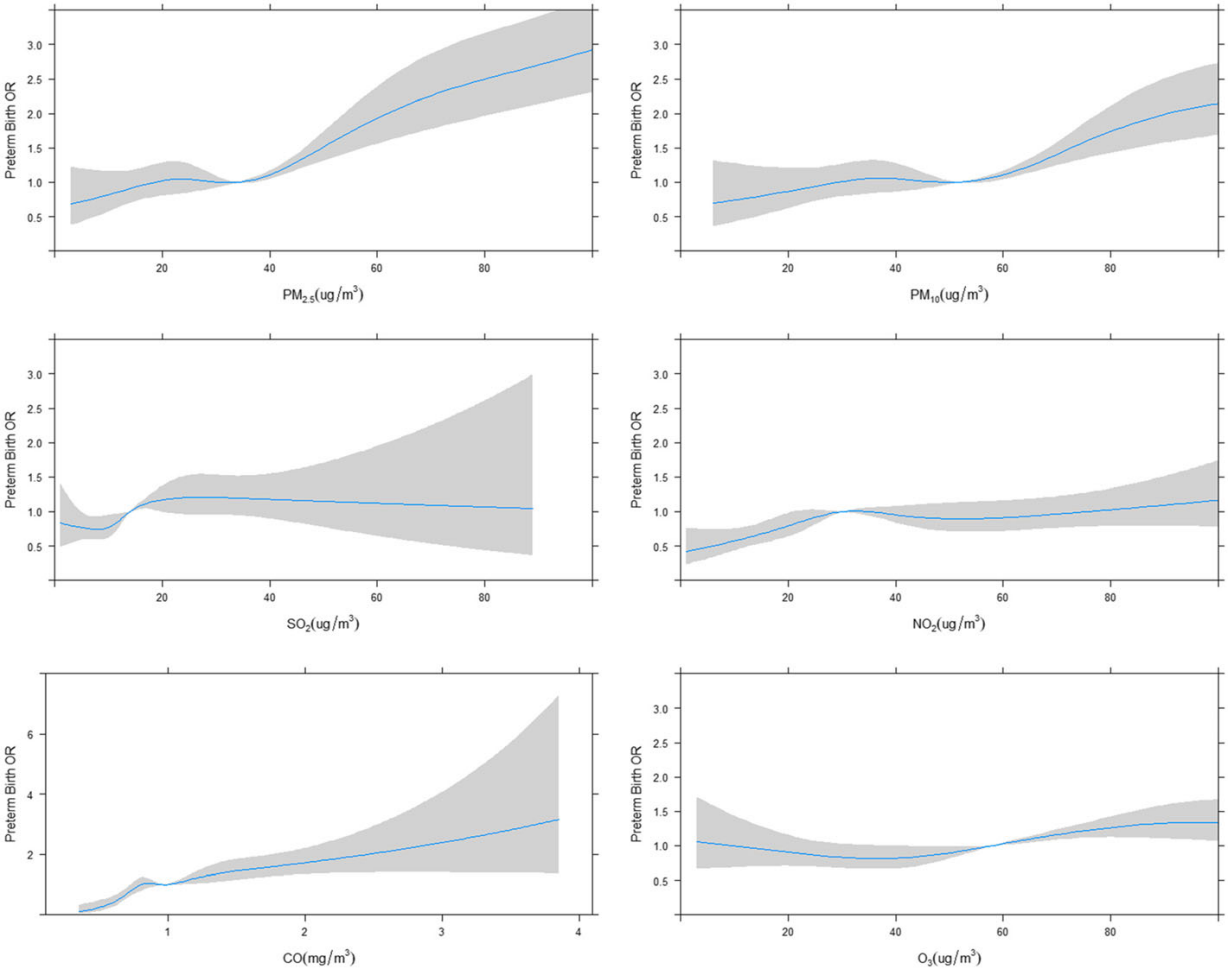
Concentration-response relationships between air pollutants and preterm birth

**Fig. 3:**
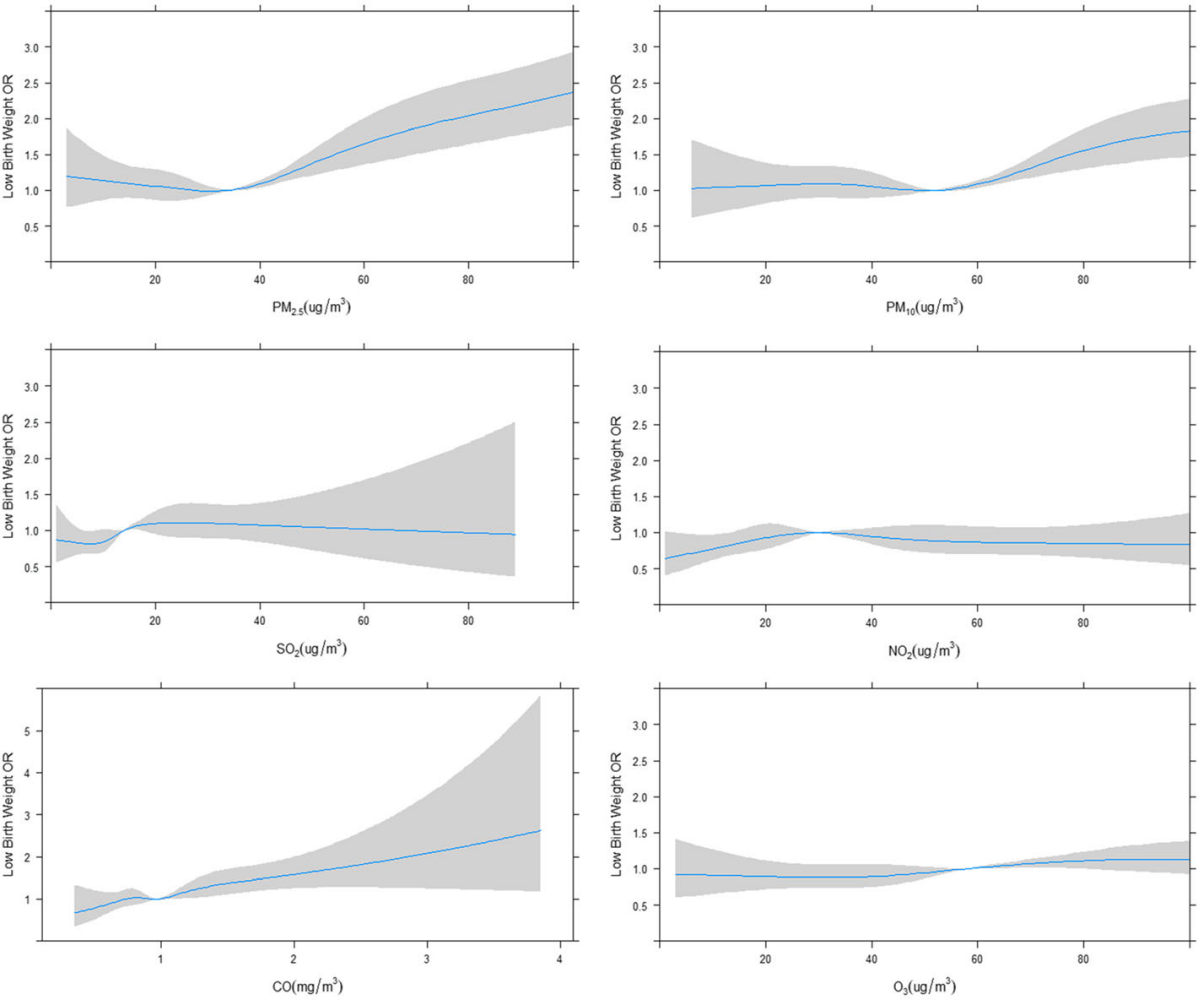
Concentration-response relationships between air pollutants and low birth weight

Figures 2 and 3 present the approximate linear relationships between air pollutants and preterm birth/low birth weight. Basic on the AIC statistics, 3 degrees of freedom were selected to represent the models. It was found from Figs. 2 and 3 that, as the concentrations of PM_2.5_, PM_10_, NO_2_, CO, and O_3_ above the knot locations increased, the risks of preterm birth/low birth weight increased as a whole. As shown in Table 4, after controlling for the impact of maternal age, education level, occupation, registered residence, gestational age, infant sex, childbirth time, month of conception, and parity, the increases in the risk of preterm birth were associated with each 10 μg/m^3^ increment in the exposure to PM_2.5_ (OR 1.043, 95% CI 1.01–1.09) and PM_10_ (OR 1.039, 95% CI 1.01~1.14) during the first trimester, indicating 4.3 and 3.9% increased risk of preterm birth, respectively. In addition, significant associations were found for preterm birth with PM_2.5_ (OR 1.038, 95% CI 1.01~1.12), PM_10_ (OR 1.024, 95% CI 1.02~1.09), SO_2_ (OR 1.081, 95% CI 1.01~1.29), and O_3_ (OR 1.016, 95% CI 1.004~1.35) during the third trimester. Moreover, the increase in the risks of low birth weight was associated with each 10 μg/m^3^ increment in NO_2_ (OR 1.124, 95% CI 1.02–1.24) during the second trimester and with each 100 μg/m^3^ increment in CO (OR 1.063, 95% CI 1.00~1.14) during the first trimester. For the entire pregnancy, the odds ratios of preterm birth for a 10 μg/m^3^ increase in PM_2.5_ and PM_10_ were 1.007 (95% CI 1.01–1.08) and 1.038 (95% CI 1.01–1.07), respectively. And the odds ratios of low birth weight for a 10 μg/m^3^ increase in PM_2.5_ and PM_10_ were 1.028 (95% CI 1.00–1.06), 1.018 (95% CI 1.01–1.04) and for a 100 μg/m^3^ increase in CO was 1.340 (95% CI 1.04–1.73), respectively.

**Table 4:** Associations between pregnancy exposure to air pollutions and preterm birth/low birth weight

	Preterm birth				Low birth weight			
	First trimester	Second trimester	Third trimester	Whole pregnancy	First trimester	Second trimester	Third trimester	Whole pregnancy
PM_2.5_								
OR	1.043^a^	1.056	1.038^a^	1.007^a^	1.063	1.061	0.925	1.028^a^
95% CI	1.01~1.09	0.98~1.14	1.01~1.12	1.01~1.08	0.98~1.15	0.99~1.13	0.86~0.99	1.00~1.06
PM_10_								
OR	1.039^a^	1.031	1.024^a^	1.038^a^	0.967^a^	1.046	0.937^a^	1.018^a^
95% CI	1.01~1.14	.96~1.10	1.02~1.09	1.01~1.07	0.91~1.03	0.99~1.11	0.88~0.99	1.01~1.04
SO_2_								
OR	0.990	1.112	1.081^a^	1.047	0.907	0.927^a^	1.019	1.007
95% CI	0.82~1.19	0.89~1.38	1.01~1.29	0.95~1.15	0.77~1.07	0.89~0.96	0.98~1.05	0.99~1.02
NO_2_								
OR	1.130	1.078	0.970	1.051	0.916	1.124^a^	0.896	1.039
95% CI	0.98~1.30	0.96~1.21	0.88~1.06	0.99~1.11	0.84~1.00	1.02~1.24	0.83~1.10	0.99~1.09
CO								
OR	1.059	0.839^a^	0.991	1.276	1.063^a^	0.845^a^	0.974	1.340^a^
95% CI	0.98~1.14	0.76~0.92	0.92~1.06	0.97~1.68	1.00~1.14	0.77~0.92	0.91~1.04	1.04~1.73
O_3_								
OR	0.891	0.932	1.016^a^	1.000	0.895	0.979	1.023	0.999
95% CI	0.76~1.04	0.89~1.06	1.004~1.35	0.99~1.01	0.778~1.03	0.87~1.10	0.889~1.18	0.99~1.00

Note: ^a^ *p*<0.05

More specifically, the associations between PM_2.5_, PM_10_, NO_2_, CO and preterm birth in the second month were found, and the ORs were 1.038 (95% CI 1.01–1.07), 1.021 (95% CI 1.01–1.04), 1.043 (95% CI 1.01–1.08) and 1.069 (95% CI 1.001–1.14), respectively (see Table 5). The associations were also observed with exposure to PM_2.5_, PM_10_ and O_3_ in the eighth month (*p* < 0.05). The ORs of premature birth for a 10 μg/m^3^ increase in NO_2_ in the last month was 1.034 (95% CI 1.00–1.07). Additionally, for each 10 μg/m^3^ increase, the resulting ORs of low birth weight were 1.059 (95% CI 1.02–1.10) for PM_2.5_, 1.090 (95% CI 1.03–1.15) for PM_10_, 1.328 (95% CI 1.01–1.75) for SO_2_, 1.185 (95% CI 1.06–1.32) for NO_2_, and 1.108 (95% CI 1.03–1.19) for O_3_ in the first month; and for each 100 μg/m^3^ increase, the ORs was 1.117 (95% CI 1.05–1.19) for CO. And the associations between PM_2.5_, PM_10_, NO_2_, O_3_ and low birth weight in the last month were also found (OR 1.082, 95% CI 1.01~1.17; OR 1.063, 95% CI 1.01~1.13; OR 1.030, 95% CI 1.01~1.15; OR 1.106, 95% CI 1.03~1.12) (see Table 6).

**Table 5:** Associations between preterm birth and pregnancy exposure to air pollutions during each month

	The first month	The second month	The third month	The fourth month	The fifth month	The sixth month	The seventh month	The eighth month	The ninth month	The tenth month
PM_2.5_										
OR	1.006	1.038^a^	1.001	0.991	1.002	0.995	1.010	1.028^a^	0.992	1.021
95% CI	0.99~1.01	1.01~1.07	0.98~1.02	0.97~1.01	0.98~1.02	0.98~1.01	0.99~1.03	1.01~1.05	0.97~1.01	0.99~1.04
PM_10_										
OR	1.006	1.021^a^	0.993	0.989	1.000	0.999	1.007	1.012^a^	0.990	1.008
95% CI	0.98~1.02	1.01~1.04	0.99~1.01	0.97~1.00	0.99~1.01	0.99~1.01	0.99~1.02	1.00~1.02	0.98~1.00	0.99~1.02
SO_2_										
OR	1.052	1.018	.920^a^	1.002	1.035	0.998	0.987	1.019	0.995	1.033
95% CI	0.99~1.12	0.97~1.07	0.88~0.96	0.95~1.05	0.98~1.09	0.95~1.05	0.94~1.03	0.98~1.06	0.96~1.04	0.99~1.07
NO_2_										
OR	1.032	1.043^a^	0.962^a^	1.010	1.013	1.016	0.986	1.021	1.000	1.034^a^
95% CI	0.99~1.08	1.01~1.08	0.93~0.99	0.97~1.05	0.98~1.06	0.98~1.05	0.95~1.02	0.98~1.06	0.97~1.03	1.00~1.07
CO										
OR	1.905^a^	1.069^a^	0.970	0.906^a^	0.887	0.902^a^	0.924	0.971	0.990	1.001
95% CI	1.02~1.17	1.001~1.14	0.91~1.04	0.84~0.98	0.81~0.97	0.84~0.97	0.87~0.98	0.91~1.03	0.931~1.05	0.93~1.08
O_3_										
OR	1.004	1.000	0.999	0.934	1.006	0.958	1.023	1.103^a^	0.987	1.007
95% CI	0.93~1.09	0.99~1.00	0.99~1.00	0.86~1.01	0.94~1.08	0.90~1.01	0.96~1.08	1.03~1.18	0.89~1.04	0.99~1.02

Note: ^a^ *p*<0.05

**Table 6:** Associations between low birth weight and pregnancy exposure to air pollutions during each month

	The first month	The second month	The third month	The fourth month	The fifth month	The sixth month	The seventh month	The eighth month	The ninth month	The tenth month
PM_2.5_										
OR	1.059^a^	1.046	0.982	0.998	1.004	0.899	0.951	0.947	0.990	1.082^a^
95% CI	1.02~1.10	0.98~1.11	0.91~1.06	0.99~1.01	0.99~1.02	0.84~0.96	0.89~1.01	0.89~1.01	0.96~1.01	1.01~1.17
PM_10_										
OR	1.090^a^	1.035	0.988	0.989	1.002	0.921	0.964	0.965	1.063^a^	1.063^a^
95% CI	1.03~1.15	0.98~1.08	0.98~1.00	0.98~1.00	0.99~1.01	0.87~1.00	0.92~1.01	0.92~1.01	1.00~1.13	1.01~1.13
SO_2_										
OR	1.328^a^	0.957	0.980	1.026	0.968	0.951^a^	0.981	0.992	1.124	1.124
95% CI	1.01~1.75	0.92~1.07	0.95~1.02	0.98~1.07	0.93~1.01	0.91~0.99	0.94~1.02	0.81~1.21	0.88~1.44	0.88~1.43
NO_2_										
OR	1.185^a^	1.137^a^	1.036	0.999	0.978	0.995	0.968^a^	0.918	1.010	1.030^a^
95% CI	1.06~1.32	1.02~1.27	0.91~1.17	0.87~1.15	0.95~1.01	0.97~1.02	0.94~0.99	0.83~1.02	0.99~1.03	1.01~1.15
CO										
OR	1.117^a^	1.038	0.978	0.930^a^	0.862^a^	0.886^a^	0.924^a^	0.942^a^	0.960	1.018
95% CI	1.05~1.19	0.97~1.11	0.92~1.04	0.87~0.99	0.79~0.94	0.83~0.95	0.87~0.98	0.89~0.99	0.90~1.02	0.95~1.09
O_3_										
OR	1.108^a^	0.999	0.999	0.885	0.984	0.993	1.084^a^	1.000	0.907	1.106^a^
95% CI	1.03~1.19	0.98~1.00	0.99~1.00	0.82~0.96	0.92~1.05	0.94~1.05	1.02~1.15	0.93~1.07	0.84~1.00	1.03~1.12

Note: ^a^ *p*<0.05

## Discussion

This study investigated the association between air pollutions (PM_2.5_, PM_10_, SO_2_, NO_2_, CO, O_3_) and preterm birth/low birth weight. The results showed that after controlling for the impact of confounding factors, there were significant associations between preterm birth and PM_2.5_, PM_10_, SO_2_, NO_2_, and O_3_, especially during the first trimester and the third trimester, which were consistent with the previous studies [18–20]. Olsson et al. (2013) indicated that the risk of preterm birth could be increased with rising O_3_ concentration during the early pregnancy [18]. Cheng et al. (2016) found that exposure to high concentrations of PM_2.5_ in the third trimester might increase the risk of preterm birth, especially in the half a month before delivery [20]. Exposure to PM_10_ also affected on preterm birth in the late pregnancy, especially in the seventh and ninth month of pregnancy [21]. Additionally, Leem et al. (2006) also found that exposure to SO_2_ in the late pregnancy was statistically significant for Percutaneous Transluminal Dilatation (PTD) patients [22]. Moreover, the increased concentration of SO_2_ during the third trimester increased the risk of preterm birth, and this relationship was statistically significant [23].

On the other hand, the significant associations were found for low birth weight with PM_2.5_, PM_10_, SO_2_, NO_2_, O_3_, CO in the first month and with PM_2.5_, PM_10_, NO_2_, O_3_ in the last month. The effects of air pollution on low birth weight also were found from the previous studies [24]. For example, Chen et al. (2000) indicated that exposure to PM_10_ in the late pregnancy could predict the neonatal weight after controlling for baby gender, the pregnant women’s age, living area, ethnic, education, drugs and alcohol use. For every 10 μg/m^3^ increase in PM_10_ concentration of 24 h during the late trimester, the weight of newborn was reduced by 11 g [24]. Dugandzic et al. (2006) collected the pregnant women data within 25 km from the air monitoring station at the Ministry of Health of Nova Scotia in Canada from 1988 to 2000, and found the higher risk effect of exposure to higher SO_2_ and PM_10_ concentrations during early pregnancy on low birth weight using the multiple regression models [25]. Gouveia et al. (2004) found that if pregnant women were exposed to CO in the early pregnancy, an increase in the mean concentration of 1 μm would reduce the weight of newborn by 23 g [26]. An interquartile of exposure to NO_2_, CO, PM_10_ and PM_2.5_ during pregnancy increased, and birth weight decreased by 8.9 g, 16.2 g, 8.2 g and 14.7 g, respectively [27]. Additionally, for each 50 μg/m^3^ increase in concentration, the OR value of the effect of exposure to SO_2_ at early pregnancy on low birth weight was 1.20, and the corresponding 95% CI was from 1.11 to 1.30 [28].

Therefore, the present study demonstrated that the early and late pregnancy might be the critical period of preterm birth and low birth weight caused by PM_2.5_, PM_10_, NO_2_, SO_2_, CO, O_3_ pollutions, and further confirmed the previous reports on the adverse effect of air pollution on preterm birth and low birth weight. These results suggested that pregnant women should reduce or avoid exposure to air pollutants during pregnancy, especially in the early and late stages of pregnancy.

This study has some limitations that merit future improvements. First, the number of monitoring sites in each city of Guangdong province is different and individual cities has only four or five monitoring sites, which might lead to incomplete monitoring data. Second, we assumed that the pollution levels were homogeneous for every resident in the present study. People in some areas inevitably expose to high pollution, while others are relatively low. Therefore, it is valuable to investigate the differences in adverse pregnancy outcomes between the areas with the highest levels and the lowest levels of air pollution in the future. Third, some other important factors associate with pregnancy outcomes are not considered in this study, such as social economic status, smoking, altitude, etc. Last, but not least, another line of research worth considering is to explore the interactions of various pollutants and other influencing factors, and the influence of two or more pollutants on preterm birth and low birth weight.

## Conclusions

This study provides further evidence for the relationships between air pollutions and preterm birth/low birth weight. The risks of preterm birth increase for each 10 μg/m^3^ increase in PM_2.5_, PM_10_ during the first trimester and in PM_2.5_, PM_10_, SO_2_, O_3_ during the third trimester. The increase in the risk of low birth weight is associated with PM_2.5_, PM_10_, NO_2_, and O_3_ in the first month and the last month. Additionally, the current study has found that the concentrations of O_3_ in September and October are relatively high, thus it is strongly recommended that pregnant women in Guangdong should avoid pregnancy during the two months with high O_3_ concentrations. Finally, public policies and guidelines for maternal health should be improved to protect women from the risks of preterm birth and low birth weight due to air pollution.

## Abbreviations

95% CIs: 95% confidence intervals
AIC: Akaike Information Criterion
CO: Carbon monoxide
*df*: degree of freedom
NO_2_: Nitrogen dioxide
O_3_: Ozone
ORs: odds ratios
PM_10_: Particulate matter of less than 10 μm in aerodynamic diameter
PM_2_: Particulate matter of less than 2.5 μm in aerodynamic diameter
PTD: Percutaneous Transluminal Dilatation
SO_2_: Sulfur dioxide
TSP: Total suspended particle
